# Early-pregnancy ALT, AST, and GGT in relation to gestational diabetes: a GGT-focused association pattern

**DOI:** 10.3389/fendo.2026.1848423

**Published:** 2026-07-24

**Authors:** Zhiheng Wang, Yan Wang, Jiawei Luo, Fangui Zhao, Chunmei Ying

**Affiliations:** 1 Clinical Laboratory, Obstetrics & Gynecology Hospital of Fudan University, Shanghai Key Laboratory of Reproduction and Development, Shanghai Key Laboratory of Female Reproductive Endocrine Related Diseases, Shanghai, China; 2 Clinical Laboratory, Obstetrics & Gynecology Hospital of Fudan University, Yangtze River Delta Integration Demonstration Zone (Qingpu), Shanghai, China

**Keywords:** alanine aminotransferase, aspartate aminotransferase, gestational diabetes mellitus, hepatic enzyme interdependence, γ-glutamyl transferase

## Abstract

**Aims:**

To explore the associations of early-pregnancy alanine aminotransferase (ALT), aspartate aminotransferase (AST), and γ-glutamyl transferase (GGT) with gestational diabetes mellitus (GDM) risk, and to examine the statistical interdependencies among these enzymes in relation to GDM.

**Methods:**

This retrospective cohort included 9,077 singleton pregnancies. Multivariate logistic regression was used to examine the associations of ALT, AST, and GGT with GDM. Statistical mediation models (PROCESS Macro Model 4) assessed whether the association of ln-ALT and ln-AST with GDM could be statistically accounted for by ln-GGT. Stratified analyses by ln-ALT quartiles examined whether the negative association of ln-AST (observed after adjustment for other enzymes) varied across levels of hepatocellular injury. Moderated mediation analysis (PROCESS Macro Model 58) examined whether age, pre-pregnancy BMI, or gestational age at sampling modified the observed associations. All models were adjusted for prespecified covariates.

**Results:**

In regression analyses, ln-GGT showed the strongest association with GDM (aOR 1.514–1.640, all *P* < 0.001). ln-ALT was associated with GDM in the single-enzyme model (*P* = 0.016) but was attenuated after ln-GGT adjustment (*P* = 0.801). ln-AST showed no association in the single-enzyme model (*P* = 0.982) but demonstrated a negative association after co-adjustment (aOR 0.494–0.738, *P* < 0.05). Mediation analysis indicated that the association of ln-ALT with GDM was largely accounted for by ln-GGT (indirect effect β=0.1754, 95% CI 0.0986–0.2527). ln-AST demonstrated contrasting statistical associations: a positive indirect component via ln-GGT (indirect effect β=0.2797, 95% CI 0.1770–0.3815) and a negative direct association (β=−0.3041, *P* = 0.037). Stratification by ln-ALT quartiles showed that the negative association of ln-AST was statistically significant only in quartiles 3–4. BMI positively, and gestational age negatively, modified the associations between ALT/AST and GGT (all *P* < 0.001).

**Conclusions:**

Early-pregnancy hepatic enzymes and GDM exhibit a GGT-focused interdependent association pattern: the association of ALT with GDM is largely accounted for by GGT, whereas AST demonstrates contrasting statistical associations—a positive indirect component via GGT and a negative direct association. This descriptive framework offers one potential statistical account for heterogeneous observational findings; prospective and experimental studies are needed to validate these patterns.

## 1 Introduction

The liver, as a central organ in glucose metabolism, is closely linked to the risk of gestational diabetes mellitus (GDM) (1–5). Early-pregnancy serum hepatic enzymes—including alanine aminotransferase (ALT), aspartate aminotransferase (AST), and γ-glutamyl transferase (GGT)—have been extensively investigated as potential biomarkers for GDM prediction. However, a persistent scientific puzzle remains: GGT consistently exhibits robust associations with GDM across diverse populations (4, 6), whereas the associations of ALT and AST with GDM have proven remarkably heterogeneous, with some studies identifying them as significant risk factors and others reporting weak or null associations (4–8). Of note, a Mendelian randomization study highlights this puzzle: it identified ALT as a causal determinant of GDM yet found no causal effect of GGT (4). This discordance suggests that the enzyme-GDM associations may be interrelated rather than independent, and that inconsistent findings across studies may partly reflect the statistical interdependence of these biomarkers.

Previous studies, while recognizing the distinct biological functions of these three enzymes, have largely treated them as parallel, independent variables without exploring their potential interdependencies. Although all three enzymes originate from the liver, their biological functions differ substantially: ALT is predominantly cytosolic and serves as a specific marker of hepatocellular integrity (9, 10); AST exists as both cytosolic and mitochondrial isoforms, with the mitochondrial component (GOT2) directly participating in the malate-aspartate shuttle and cellular energy metabolism (10–13); GGT, meanwhile, is a critical enzyme in glutathione metabolism and serves as a sensitive indicator of oxidative stress and hepatobiliary dysfunction (14–17). This functional heterogeneity suggests that these enzymes may occupy distinct roles within an interconnected physiological system, warranting investigation beyond isolated analyses. Of particular relevance, oxidative stress is a key pathophysiological pathway in GDM (18, 19). As a sensitive marker of oxidative stress and hepatobiliary function, GGT may therefore be biologically positioned to integrate metabolic signals relevant to GDM risk.

Accordingly, we hypothesize that inconsistent findings across prior studies may reflect statistical interdependencies among hepatic enzymes—including potential statistical interactions—rather than independent parallel associations. Given GGT’s role in oxidative stress, we explored whether it might statistically account for the associations of ALT and AST with GDM. We examined this hypothesis in a retrospective cohort of 9,077 singleton pregnancies using an exploratory analytical approach.

## 2 Methods

### 2.1 Study participants

This retrospective cohort included pregnant women who delivered at Obstetrics and Gynecology Hospital of Fudan University (Shanghai, China) between October 2018 and January 2022. The study was approved by the Ethics Committee (No. 2020-49) with waived informed consent due to retrospective anonymized data.

Inclusion: Singleton live birth, Chinese nationality, 75-g OGTT at 24–28 weeks with complete results, hepatic function testing (ALT, AST, GGT) before 20 weeks. For multiple pregnancies, only the first eligible pregnancy was included.

Exclusion: Multiple gestation, pre-existing diabetes, hepatic disorders (>2× upper limit of normal [ULN]), thyroid dysfunction (TSH >10 mIU/L or FT4 >40 pmol/L), chronic nephritis, metabolic disorders, fetal abnormalities, assisted reproductive technology, non-Chinese, OGTT <24 weeks (to exclude potential interference from early-onset glycemic dysregulation), missing covariates.

Of the initial 18,973 pregnancies, 9,077 were included in the final cohort (**Supplementary Figure 1**). Among the exclusions, 2,792 were actively excluded based on pre-specified criteria, and 7,104 were excluded due to incomplete data (5,344 lacked valid 75-g OGTT results at 24–28 weeks, 978 lacked early-pregnancy liver enzyme measurements, and 782 had missing covariate data). As a regional tertiary referral center specializing in complicated pregnancies, our hospital receives numerous patients transferred in the second or third trimester. This leads to structural, non-random missingness of OGTT and liver enzyme data. Since OGTT (the study outcome) and hepatic enzymes (core exposure variables) are fundamental to our research hypotheses, imputation for these key variables was deemed inappropriate. The proportion of participants with missing covariate data was only 4.12% (782/18,973), making complete-case analysis a reasonable and rigorous analytical choice. Accordingly, we adopted complete-case analysis for analyses.

### 2.2 Variables and outcomes

Data were extracted from electronic medical records. Laboratory parameters (ALT, AST, GGT, fasting plasma glucose [FPG], triglycerides, creatinine, and thyroid-stimulating hormone [TSH] were measured from early-pregnancy (<20 weeks) fasting samples. Covariates included age, parity, pre-pregnancy BMI, gestational age at sampling (GA, days), and family history of diabetes. GDM was diagnosed by 75-g OGTT at 24–28 weeks using IADPSG criteria (fasting ≥5.1, 1-hour ≥10.0, or 2-hour ≥8.5 mmol/L).

### 2.3 Statistical analysis

#### 2.3.1 Analytical strategy overview

To examine our hypothesis, we adopted a stepwise analytical strategy within the retrospective cohort of 9,077 singleton pregnancies. First, we explored patterns of mutual statistical adjustment among hepatic enzymes using single-enzyme and mutually adjusted regression models. Second, we tested for potential multiplicative and additive interactions between enzyme pairs. Third, we performed statistical mediation analysis (PROCESS Macro Model 4) to decompose the associations of ln-ALT and ln-AST with GDM via ln-GGT. Finally, we examined whether age, pre-pregnancy BMI, or gestational age at sampling modified the observed association patterns using moderated mediation analysis (PROCESS Macro Model 58).

#### 2.3.2 Detailed procedures

All analyses were performed using SPSS 29.0, PROCESS macro 5.0, and R 4.5.2. Two-tailed *P* < 0.05 was considered significant. Continuous and categorical variables were compared using t-test/Mann-Whitney U and chi-square tests, respectively, after normality assessment. ALT, AST, and GGT were natural log-transformed due to right-skewed distributions.

Model validation confirmed linearity for all model variables including ALT, AST, and GGT (restricted cubic spline, all non-linearity *P* > 0.05), and absence of problematic multicollinearity specifically among the three hepatic enzymes and across all covariates (maximum VIF = 3.306). These diagnostics support the validity of the linear interaction and mediation models employed.

All regression models were adjusted for age, BMI, GA, FPG, triglycerides, TSH, creatinine, parity, ethnicity, and family history of diabetes. Multivariable logistic regression with sequential enzyme inclusion examined independent associations and mutual influences. Multiplicative and additive interactions (RERI, S) were tested between enzyme pairs.

Following recent exploratory applications of statistical decomposition in observational studies (20–23), we employed PROCESS Macro Model 4 with 5,000 bootstrap resamples to decompose the associations among ALT, AST, GGT and GDM. Traditional causal mediation analysis is based on four main assumptions: temporal precedence, no unmeasured confounding, correct model specification, and no measurement error (24, 25). Except for temporal precedence, the other three assumptions were reasonably supported in our study. Specifically, we adjusted for a broad set of prespecified covariates (age, pre−pregnancy BMI, gestational age at sampling, fasting plasma glucose, triglycerides, TSH, creatinine, parity, ethnicity, and family history of diabetes). Restricted cubic splines confirmed linearity for all enzymes (all P > 0.05). Neither multiplicative nor additive interactions were significant (see Results). The maximum variance inflation factor was 3.306, indicating no problematic multicollinearity. All biochemical measurements were performed under standardized quality control in a tertiary hospital clinical laboratory. Because ALT, AST, and GGT were measured in the same early−pregnancy blood sample, the temporal precedence assumption was not met. Therefore, we do not interpret the decomposed indirect and direct statistical components as causal pathways; rather, they are descriptive statistical partitions of shared and independent variations among the three liver enzymes.

### 2.3.3 Exploratory stratified and moderated analyses

Stratified analyses by ln-ALT quartiles examined whether the negative association of ln-AST (observed after adjustment for other enzymes) varied across levels of hepatocellular injury. Moderated mediation analysis (PROCESS Model 58) was used to examine whether age, pre−pregnancy BMI, or gestational age at sampling modified the indirect associations of ALT and AST with GDM via GGT. Conditional indirect effects were estimated at the 16th, 50th, and 84th percentiles of the moderators.

Sensitivity analyses (outlier exclusion, covariate adjustment strategies, Box-Cox transformation) confirmed the robustness of findings.

## 3 Results

### 3.1 Baseline characteristics

The cohort included 9,077 women (847 GDM, 9.3%). Compared with NGT, women with GDM were older, had higher pre-pregnancy BMI, higher FPG and triglycerides, were less likely to be nulliparous, and were more likely to have a family history of diabetes (all *P* < 0.05). ALT and GGT were higher in the GDM group (both *P* < 0.001); ln-transformed values showed similar patterns. AST did not differ. **Table 1** presents full characteristics.

**Table 1:** Baseline Characteristics of the Study Population.

Variable	GDM (n=847)	NGT (n=8230)	*P*-value
Demographics			
Age (years)	32.10±3.96	30.74±3.92	<0.001
Parity, n (%)			
Nulliparity	637 (75.2%)	6461 (78.5%)	0.027
Multiparity	210 (24.8%)	1769 (21.5%)	
Ethnic group, n (%)			
Han	831 (98.1%)	7965 (96.8%)	0.033
Minority	16 (1.9%)	265 (3.2%)	
Anthropometric and Metabolic Indicators			
Pre-pregnancy BMI (kg/m²)	22.33±3.11	21.06±2.76	<0.001
Family history of diabetes, n (%)			
Yes	134 (15.8%)	698 (8.5%)	<0.001
No	713 (84.2%)	7532 (91.5%)	
24–28 Week OGTT (mmol/L)			
Fasting glucose	4.60±0.56	4.17±0.32	<0.001
1-hour glucose	9.98±1.42	7.11±1.36	<0.001
2-hour glucose	8.41±1.51	6.07±1.03	<0.001
Early Pregnancy (< 20 Weeks) Indicators			
GA (days)	74.71±14.90	75.33±15.57	0.273
FPG (mmol/L)	4.57±0.36	4.46±0.31	<0.001
Triglycerides (mmol/L)	1.35 (0.99, 1.78)	1.16 (0.90, 1.51)	<0.001
TSH (mIU/L)	1.54±1.04	1.54±1.11	0.983
Creatinine (μmol/L)	43.80±6.06	43.80±5.71	0.984
ALT (U/L)	15.00 (11.00, 22.00)	13.00 (10.00, 19.00)	<0.001
ln-ALT	2.77±0.58	2.66±0.53	<0.001
AST (U/L)	16.00 (14.00, 20.00)	17.00 (14.00, 20.00)	0.729
ln-AST	2.85±0.29	2.84±0.27	0.631
GGT (U/L)	15.00 (11.00, 22.00)	13.00 (10.00, 17.00)	<0.001
ln-GGT	2.75±0.50	2.59±0.43	<0.001

Data are mean ± SD (normally distributed continuous variables), median (IQR) (non-normally distributed), and n (%) (categorical variables). Between-group comparisons used t-test/Mann-Whitney U test (continuous) and chi-square test (categorical). IQR, interquartile range.

### 3.2 Independent associations of ALT/AST/GGT with GDM risk and mutual influences

To elucidate interrelationships among ALT/AST/GGT, we employed multivariable logistic regression with sequential inclusion of single enzymes, two-enzyme combinations, and all three enzymes (**Table 2**). The single-enzyme models (Model 1) showed that both ln-GGT (adjusted odds ratio [aOR] 1.522, 95% confidence interval [CI] 1.294–1.790) and ln-ALT (aOR 1.179, 95% CI 1.031–1.347) were positively associated with GDM, whereas ln-AST showed no significant association (aOR 0.997, 95% CI 0.768–1.295). However, joint analyses revealed complex statistical interdependencies among these enzymes

**Table 2:** Association of Early Pregnancy Serum ALT/AST/GGT With GDM: Single, Pairwise, and Joint Regression Analysis.

Model	Variables	OR (95% CI)	P-value
**Univariable**	ln-ALT	1.440 (1.271–1.631)	<0.001
	ln-AST	1.069 (0.826–1.384)	0.610
	ln-GGT	2.097 (1.811–2.430)	<0.001
**Multivariable**			
Model 1: Single-enzyme	ln-ALT	1.179 (1.031–1.347)	0.016
	ln-AST	0.997 (0.768–1.295)	0.982
	ln-GGT	1.522 (1.294–1.790)	<0.001
Model 2: ALT + AST	ln-ALT	1.585 (1.266–1.986)	<0.001
	ln-AST	0.494 (0.322–0.760)	0.001
Model 3: ALT + GGT	ln-ALT	0.980 (0.836–1.148)	0.801
	ln-GGT	1.542 (1.273–1.867)	<0.001
Model 4: AST + GGT	ln-AST	0.738 (0.554–0.982)	0.037
	ln-GGT	1.640 (1.374–1.956)	<0.001
Model 5: Three-enzyme joint	ln-ALT	1.292 (1.011–1.650)	0.040
	ln-AST	0.525 (0.341–0.809)	0.003
	ln-GGT	1.514 (1.249–1.836)	<0.001

ALT/AST/GGT were natural log-transformed (ln-) for all analyses. Univariable models: unadjusted. Multivariable models: adjusted for age, BMI, GA, FPG, triglycerides, TSH, creatinine, parity, ethnicity, and family history of diabetes. Models: 1 (Single-enzyme): ln-ALT/ln-AST/ln-GGT entered separately; 2 (ALT+AST): ln-ALT+ln-AST; 3 (ALT+GGT): ln-ALT+ln-GGT; 4 (AST+GGT): ln-AST+ln-GGT; 5 (Three-enzyme): ln-ALT+ln-AST+ln-GGT. Abbreviations: CI, confidence interval; OR, odds ratio.

ln-GGT demonstrated the most robust association: Regardless of adjustment for ln-ALT, ln-AST, or both, ln-GGT remained significantly and positively associated with GDM (aOR range: 1.514–1.640, all *P* < 0.001), consistently exhibiting the strongest association magnitude among the three enzymes.

The association of ln−ALT with GDM was no longer significant after adjustment for ln−GGT (Model 3: aOR 0.980, 95% CI: 0.836–1.148), suggesting that the observed association of ln-ALT with GDM may be largely explained by ln-GGT.

ln-AST demonstrated a negative association in joint models: ln-AST, which showed no association in single-enzyme analysis, exhibited significant negative associations once adjusted for ln-ALT or ln-GGT, with the strongest negative association in the ALT+AST model (aOR 0.494, 95% CI: 0.322–0.760); this negative association persisted in the three-enzyme model (aOR 0.525, 95% CI: 0.341–0.809).

Collectively, these findings indicate that the three hepatic enzymes do not exhibit parallel, independent associations with GDM. GGT showed the most consistent positive association, the association of ALT was statistically explained by GGT, and AST displayed negative associations only after adjustment for other enzymes—suggesting statistical interdependencies rather than purely independent effects.

### 3.3 Interaction and mediation analysis

#### 3.3.1 No significant interactions among ALT/AST/GGT

To further examine whether the associations of ALT, AST, and GGT with GDM involve statistical interactions, multiplicative and additive interaction analyses were performed. The results showed no statistically significant multiplicative interaction terms between ln-ALT and ln-GGT, ln-AST and ln-GGT, or ln-ALT and ln-AST (*P* = 0.938, 0.677, and 0.234, respectively); the 95% confidence intervals of the additive interaction indicator RERI all included 0, and the 95% CIs of S all included 1 (**Supplementary Table 1**), indicating no significant additive interaction effects. These findings indicate that the enzyme-GDM associations do not follow simple synergistic or antagonistic interaction patterns, prompting exploration of their interdependent association structure through complementary statistical approaches.

#### 3.3.2 GGT-focused statistical decomposition of ALT/AST-GDM associations

To explore whether the interdependent associations among hepatic enzymes could be statistically decomposed, we examined whether ln-GGT statistically accounted for the associations of ln-ALT and ln-AST with GDM. We employed PROCESS Macro Model 4 (5,000 bootstrap) to decompose the ln-ALT/ln-AST-GDM associations into direct and indirect components with ln-GGT as the analytical focus (**Table 3**).

**Table 3:** Bootstrap Mediation Analysis of ln-GGT in the Association Between ln-ALT/ln-AST and GDM.

Liver Enzyme Index	Effect Type	β (Effect Value)	Bootstrap SE	95% Bootstrap CI	*P*-value	Mediation Type
ln-ALT	Direct effect	-0.0204	0.0809	-0.1790 to 0.1382	0.801	Complete mediation
	Indirect effect (via ln-GGT)	0.1754	0.0390	0.0986 to 0.2527	/	
ln-AST	Direct effect	-0.3041	0.1460	-0.5902 to -0.0180	0.037	Inconsistent mediation
	Indirect effect (via ln-GGT)	0.2797	0.0515	0.1770 to 0.3815	/	

All liver enzymes were natural log-transformed (ln-). Bootstrap analysis (5000 resamples) was used; 95% CI excluding 0 indicates significance. All models adjusted for age, BMI, GA, FPG, triglycerides, TSH, creatinine, parity, ethnicity, and family history of diabetes. CI, confidence interval; SE, standard error.

For ln-ALT, the association with GDM was largely accounted for by ln−GGT: the direct component was not statistically significant (β = −0.0204, *P* = 0.801), whereas the indirect component via ln−GGT was significantly positive (β = 0.1754, 95% bootstrap CI: 0.0986 to 0.2527).

For ln-AST, a contrasting statistical pattern emerged: a positive indirect association via ln-GGT (β = 0.2797, 95% bootstrap CI: 0.1770 to 0.3815) alongside a negative direct association (β = −0.3041, P = 0.037). This pattern aligns with the negative ln-AST-GDM association observed in joint models (Table 2). The near-null total association in single-enzyme models reflects the counterbalancing of these opposing components.

### 3.4 Stratified analysis: AST–GDM association across ALT quartiles

To examine whether the negative association of ln−AST observed in joint models varied across levels of hepatocellular injury (as reflected by ALT), we stratified the study population by ln−ALT quartiles. Multivariable logistic regression models were fitted within each stratum, simultaneously including ln−ALT and ln−AST and adjusting for age, BMI, GA, FPG, triglycerides, TSH, creatinine, parity, ethnicity, and family history of diabetes.

As shown in **Supplementary Table 2**, the adjusted odds ratios (aOR) for ln−AST were below 1 across all ln−ALT quartiles. The negative association did not reach statistical significance in the lowest two quartiles (Q1: aOR = 0.469, 95% CI: 0.177–1.242, *P* = 0.128; Q2: aOR = 0.508, 95% CI: 0.173–1.487, *P* = 0.217). In contrast, statistical significance was reached in the upper two quartiles (Q3: aOR = 0.333, 95% CI: 0.120–0.919, *P* = 0.034; Q4: aOR = 0.484, 95% CI: 0.243–0.966, *P* = 0.039). The magnitude of the negative association was larger in Q3 than in Q4.

### 3.5 Moderating effects of age, BMI, and GA in mediation analysis

BMI and GA significantly modified the associations of ALT and AST with GGT (all *P* < 0.001), with stronger associations at higher BMI and earlier gestational stages (**Supplementary Table 3**). Age showed no modification. Neither BMI nor GA modified the GGT–GDM association (*P* > 0.05).

### 3.6 Sensitivity analyses

To assess the stability of our statistical findings, we conducted three sensitivity analyses: excluding enzyme outliers, applying alternative covariate adjustment strategies, and employing Box-Cox transformation instead of natural logarithmic transformation. Results demonstrated that the patterns observed for both ALT (where the association was fully accounted for by GGT) and AST (where opposing direct and indirect components emerged) remained consistent with the primary analyses across all sensitivity scenarios (**Supplementary Tables 4–S6**), supporting the stability of the observed statistical patterns.

## 4 Discussion

This study indicates that ALT, AST and GGT are not independent markers in relation to GDM. Instead, the three enzymes present distinct statistical association patterns with GDM, with GGT serving as the statistical focal point of the decomposition. The association between ALT and GDM is largely explained by GGT, while AST shows two contrasting statistical links: an indirect association via GGT and an independent negative direct association. These findings put forward a statistical framework as a working hypothesis from observational retrospective data. It helps explain the heterogeneous results seen in previous observational studies.

### 4.1 Heterogeneous and interdependent statistical associations of hepatic enzymes with GDM

To elucidate the inconsistent findings across previous studies (4–8), we analyzed the associations between hepatic enzymes and GDM using single-enzyme and mutually adjusted regression models. Consistent with prior reports, GDM showed a strong statistical association with ln-GGT across all models (aOR: 1.514–1.640, all *P* < 0.001) *(*4–8). The significant association between ln-ALT and GDM observed in the single-enzyme model disappeared after adjusting for ln-GGT (aOR 0.980, 95% CI 0.836–1.148). By contrast, ln-AST presented a negative statistical association with GDM only in mutually adjusted models (aOR range: 0.494–0.738). Notably, these enzymes did not exhibit problematic multicollinearity (maximum VIF = 3.306); therefore, the observed attenuation of ALT after GGT adjustment and the emergence of AST’s negative component reflect statistical overlap in their associations with GDM rather than collinearity. These findings raise the hypothesis that statistical overlap among ALT, AST, and GGT may partly account for the heterogeneous associations observed in prior literature, although other methodological and population-specific factors cannot be excluded.

GGT is centrally involved in glutathione metabolism and is a sensitive indicator of oxidative stress and hepatobiliary dysfunction (14–17). In contrast, ALT and AST primarily reflect hepatocellular integrity and energy metabolism (9, 12, 13, 26–29). These distinct biological roles provide a rationale for selecting GGT as the analytical focus—based on its relevance to oxidative stress pathways implicated in GDM pathogenesis (18, 19), rather than solely on its statistical association strength. This selection does not imply that GGT mediates biological causation, but it grounds the statistical decomposition in a biologically informed framework.

### 4.2 Statistical decomposition of ALT/AST–GDM associations with GGT as analytical focus

Given that the divergent associations among hepatic enzymes suggested statistical interdependence rather than simple effect modification, we first tested for multiplicative and additive interactions. The null interaction findings (multiplicative interaction *P* > 0.05; additive interaction RERI 95% CI spanning 0) were consistent with the absence of synergistic or antagonistic relationships and led us to explore whether their interdependent associations could be statistically decomposed through exploratory mediation analysis.

We employed PROCESS Macro Model 4 (5,000 bootstrap) to decompose the associations of ln-ALT and ln-AST with GDM into direct and indirect components, with ln-GGT as the analytical focus. For ln−ALT, the direct component was not statistically significant (β = −0.0204, *P* = 0.801), whereas the indirect component via ln−GGT was positive and statistically significant (β = 0.1754, 95% CI: 0.0986–0.2527). These decomposition results are consistent with the regression findings and further indicate that the positive statistical association of ALT with GDM is largely accounted for by GGT. This pattern offers a possible statistical explanation for why the ALT–GDM association became non−significant after adjustment for GGT, and more generally, for the instability of ALT across different models.

These findings also offer a possible explanation for the heterogeneous results in the literature, where ALT–GDM associations have ranged from positive to null. Single−enzyme models, as used in most previous studies, may capture the shared variation between ALT and GGT without separating the two (4–7). Regarding the discrepancy between observational and Mendelian randomization evidence (suggesting a genetic association for ALT but not GGT) (4), our findings cannot directly resolve this paradox.

For ln-AST, the mediation analysis revealed contrasting statistical components: a positive indirect association with GGT (β = 0.2797, 95% CI: 0.1770–0.3815) alongside a negative direct association (β = −0.3041, *P* = 0.037). The near-null total association of AST in single-enzyme models reflected counterbalancing opposing components: a positive indirect effect via GGT and a negative direct effect. This statistical pattern may offer one possible explanation for the null and inconsistent findings of AST in previous studies (4–6, 22). Differences in covariate adjustment and sample characteristics may potentially change the relative magnitude of these opposing components.

The overall mediation patterns for ALT and AST remained consistent across three sensitivity analyses, including outlier exclusion, alternative covariate adjustment and Box-Cox transformation (**Supplementary Tables 4–S6**), which supports the robustness of our statistical findings.

All interpretations presented herein are statistical hypotheses derived from retrospective observational data with contemporaneous enzyme measurements; they do not infer biological causation or temporal sequence. Several alternative mechanisms may contribute to the observed patterns. First, shared variance among the three enzymes means that the decomposition of ALT’s association into a GGT−shared component is partly a mathematical consequence of their inherent correlations. Second, overadjustment is possible when mutually adjusting for correlated biomarkers: the negative direct association of AST emerged only after including ALT and GGT, which could reflect statistical competition rather than true biological suppression. Third, residual confounding (e.g., by alcohol intake) cannot be fully excluded, particularly for GGT. Fourth, collider bias may arise when conditioning on correlated enzymes in mutually adjusted models. These alternative explanations do not invalidate our decomposition, but they underscore that the GGT−focused pattern should be understood as a statistical description rather than evidence of a unique or biologically causal structure. Our results are therefore hypothesis−generating, and future prospective studies are needed to disentangle these possibilities.

### 4.3 Stability of AST’s negative association across ALT levels

The negative statistical association of ln-AST with GDM was observed only after mutual adjustment for ln-ALT and ln-GGT. Given the known correlation between AST and ALT, a potential concern is that this pattern could stem from confounding or statistical artifacts related to hepatocellular injury. To test this possibility, we stratified participants by ln-ALT quartiles to approximate different degrees of hepatocellular injury and reassessed the AST–GDM association across strata.

If this negative association were purely an artifact driven by hepatocellular injury confounding, it would be expected to be most prominent at the lowest ALT levels and gradually weaken as ALT concentrations rose. Contrary to this expectation, the association was non-significant in Q1 and Q2 (aOR = 0.469 and 0.508, both *P* > 0.05) but reached statistical significance in Q3 and Q4 (aOR = 0.333 and 0.484, *P* = 0.034 and 0.039), with the largest effect size in Q3 and a slight attenuation in Q4. This nonlinear distribution across strata indicates that the negative association of ln-AST is unlikely to be a simple confounding artifact of hepatocellular injury.

### 4.4 Exploratory Moderation by BMI and GA

Interaction analyses explored whether age, pre-pregnancy BMI, or gestational age at sampling modified the observed statistical associations. BMI and gestational age showed statistical interactions with the associations of ALT and AST with GGT (both *P* < 0.001): the strength of these associations was greater at higher BMI and at earlier gestational weeks (**Supplementary Table 3**). Age showed no such interaction. Neither BMI nor gestational age modified the GGT–GDM association (*P* > 0.05). These exploratory findings did not change the main statistical patterns described above. This suggests that metabolic context and sampling timing may influence the observed associations, but these findings remain statistical observations requiring prospective replication.

### 4.5 Study limitations

Several limitations should be considered when interpreting these findings. First, the observational design precludes causal inference. ALT, AST, and GGT were measured contemporaneously in early pregnancy, so the temporal sequence required for causal pathways cannot be established. These results therefore represent statistical decomposition rather than biological causation. Consistent with the framework described in the Methods, the indirect statistical components largely reflect the inherent correlations among ALT, AST, and GGT rather than independent mediation processes. Prospective cohorts and experimental studies are needed to validate these findings. Second, alcohol intake was not incorporated as a covariate, owing to incomplete documentation in medical histories, potentially biasing GGT-related estimates. Given the low reported drinking rate among pregnant women in China (2.1%) (30), the resulting unmeasured confounding is likely limited, and our findings may still offer useful reference. Nevertheless, detailed alcohol assessment in future studies is warranted to clarify this potential influence.

The other limitation is that the single-center design, stringent exclusion criteria, and data completeness strategy jointly constrain generalizability. As a regional tertiary referral center, our hospital receives a large number of patients referred during the second or third trimester due to complicated conditions, which is a major source of missing 24–28 week OGTT and early-pregnancy liver enzyme data. To ensure homogeneity, we applied strict exclusion criteria (e.g., liver enzymes >2× ULN, active hepatitis, non-standard OGTT before 24 weeks), resulting in a final cohort of 9,077 participants from 18,973 initial pregnancies. Because the missingness of OGTT and liver enzyme data was structural and non-random, and covariate missing rate was low (4.12%), we used complete-case analysis rather than multiple imputation; this strategy may still introduce selection bias. To assess potential selection bias, we compared baseline characteristics between the final analytic cohort and excluded participants with complete basic covariates (n=8,834, a subset of total excluded n=9,896). Excluded participants were significantly older (31.17 ± 4.04 vs. 30.86 ± 3.95 years), had higher pre-pregnancy BMI (21.60 ± 3.20 vs. 21.18 ± 2.81 kg/m²), were more likely to be multiparity (25.10% vs. 21.80%), and had a higher prevalence of family history of diabetes (11.60% vs. 9.20%) (all P < 0.001; **Supplementary Table 7**). These characteristics indicate that the excluded population represented a higher-risk subgroup. Whether retaining these excluded higher-risk participants would strengthen, weaken, or otherwise alter the observed associations is uncertain. Consequently, these factors collectively constrain extrapolation of our findings to other settings or broader populations, and validation through multicenter, cross-population cohorts with expanded inclusion criteria is needed.

## 5 Conclusion

In summary, early−pregnancy ALT, AST, and GGT do not exhibit independent statistical associations with GDM. Their associations follow a GGT−focused pattern: the ALT–GDM association is largely accounted for by GGT, whereas AST displays a positive indirect component via GGT alongside a negative direct component. This pattern offers a possible statistical interpretation for the heterogeneous findings across observational studies. The actual causal pathways, however, require further prospective or experimental validation.

## Data Availability

The datasets presented in this article are not readily available because the clinical records contain sensitive maternal health data. Per regulations of our hospital ethics committee and institutional data governance, unrestricted public distribution of individual-level clinical datasets is forbidden, even after de-identification, unless formal data access approval is obtained. Requests to access the datasets should be directed to Prof. Chunmei Ying (E-mail: ycmzh2012@163.com). All aggregated statistical results supporting our conclusions are fully provided within the manuscript. Qualified researchers may apply for de-identified individual data by contacting the corresponding author above; data sharing will be granted after institutional ethics review and signing a confidential data use agreement.

## Supplementary material

Supplementary Figure 1 Flowchart of Study Participant Selection. Participants were excluded based on the first encountered criterion to ensure mutually exclusive categories. The order in the flowchart does not represent exact data cleaning steps but groups exclusions for clarity. Ineligible baseline criteria included OGTT before 24 weeks (outside IADPSG standard window). Abbreviations: NGT, normal glucose tolerance; OGTT, oral glucose tolerance test; ULN, upper limit of normal.
